# A small and robust active beamstop for scattering experiments on high-brilliance undulator beamlines

**DOI:** 10.1107/S160057751402829X

**Published:** 2015-02-04

**Authors:** Clement E. Blanchet, Christoph Hermes, Dmitri I. Svergun, Stefan Fiedler

**Affiliations:** a European Molecular Biology Laboratory, Hamburg Outstation c/o DESY, Notkestrasse 85, 22603 Hamburg, Germany

**Keywords:** back-scattering, radiation resistance, SAXS, small footprint, silicon PIN diode

## Abstract

A small active in-vacuum beamstop has been developed to monitor the flux of intense third-generation synchrotron X-ray beams protecting the downstream detector from the direct beam. Standard active beamstops, where a built-in diode directly absorbs the beam, have limitations in size and lifetime. In the present design, a silicon PIN diode detects the photons back-scattered from a cavity in the beamstop. This approach drastically reduces the radiation dose on the diode and thus increases its lifetime. The beamstop with a diameter of 2 mm has been fabricated to meet the requirements for the P12 bioSAXS beamline of EMBL Hamburg at PETRA III (DESY). The beamstop is in regular user operation at the beamline and displays a good response over the range of energies tested (6–20 keV). Further miniaturization of the diode is easily possible as its size is not limited by the PIN diode used.

## Abstract 2

Using an indirect detection scheme, a small-size beamstop was developed to accurately measure X-ray beam flux in a wide energy range with reduced radiation level on the electronics and significantly increased in-beam lifetime.

## 1. Introduction   

**Figure 1:**
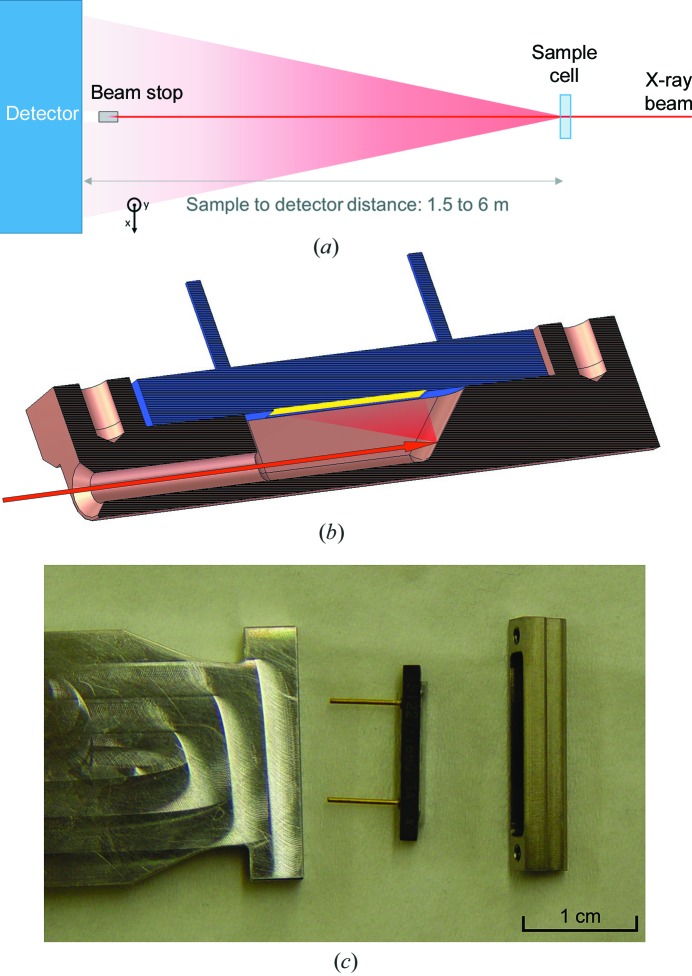
(*a*) Schematics of a SAXS experiment: the X-ray beam (red) hits the sample, photons are scattered by the sample and are collected on the detector. The transmitted direct beam is intercepted by the beamstop. (*b*) Cross-sectional drawing of the beamstop illustrating its principle. The incident beam, represented by the red arrow, enters the tungsten chamber (in orange) and hits the back of the chamber. Part of the beam is scattered on the diode (in blue). The signal produced by the photons counted on the photosensitive area (in yellow) is proportional to the incident beam intensity. (*c*) Photograph of the beamstop elements: the diode (in the center), is mounted on the profiled aluminium bar (on the left) and covered by the tungsten chamber (on the right).

The flux of the beam transmitted through a sample is measured in small-angle X-ray scattering (SAXS) experiments to accurately calibrate the collected data. This parameter is of crucial importance for the analysis of SAXS data, in particular, coming from weakly scattering samples such as biological macromolecules in solution. As the primary function of the beamstop is to absorb the intensity of the direct beam behind the sample, thus protecting the detector, the beamstop will always intersect the beam during operation and therefore constitutes a place of choice to integrate an X-ray sensor (Fig. 1 ▶ *a*). The flux of the transmitted intensity can be measured here during the exposure of the sample without disturbing the data collection.

Different approaches have been used to measure the beam flux using the beamstop: (*a*) X-ray photons can be detected on the detector through a semi-transparent beamstop (Koch *et al.*, 2003 ▶); (*b*) by a scintillating crystal, they can be transformed into visible light which is subsequently measured with a photodiode (Englich *et al.*, 2011 ▶; Kirby *et al.*, 2013 ▶); (*c*) the photo-electric effect has been successfully used to measure X-ray beam intensity in small-size beamstops (Xu *et al.*, 2010 ▶; Pan *et al.*, 2014 ▶); (*d*) direct detection on a diode also provides a convenient way to measure the flux of an X-ray beam and is commonly used in active beamstops (Ellis *et al.*, 2003 ▶; Owen *et al.*, 2009 ▶). When X-rays are absorbed by a diode, electron–hole pairs are created. The creation of one electron–hole pair needs about 3.65 eV [see *e.g.* Scholze *et al.* (2000 ▶)]. By using a reversed bias voltage or a PIN diode with a large depleted zone, recombination of the electron–hole pairs is impossible resulting in a photocurrent proportional to the X-ray photon flux.

The size of the beamstop, or more precisely the area of the projection of the beamstop onto the detector plane along the beam direction, is an important parameter because it masks out part of the detector and restricts the area where scattered/diffracted photons can be collected. For SAXS it is important to minimize this area because the signal collected in the vicinity of the direct beam carries information about the longest accessible length scales and limits the size of the particles that can be studied on the beamline. A small beamstop using a miniature PIN diode in an elegant design is described by Ellis *et al.* (2003 ▶). A similar approach was considered for the beamstop of the high-brilliance SAXS beamline P12 of the EMBL Hamburg (at PETRA III, DESY, Germany). However, initial tests showed that the miniature PIN diode (Phillips BAP64) planned to be used for the beamstop could not stand the high radiation load of the P12 undulator beam (10^13^ photons per second in 200 × 110 µm FWHM) and was rapidly degrading. This could be clearly seen by visual inspection of the diode (Fig. S1 of the supporting information^1^) and by the measurement of the photocurrent (drop of the photocurrent by a factor of 20 during 12 hours of exposure to the beam). Other models of diodes showed a similar behaviour. Adding an attenuation foil to reduce the radiation load on the diode was not considered as it leads to large variation of the beamstop response with the beam energy: at low energy, the photons are absorbed by the foil and hardly any are detected on the diode; at high energy, the foil is quasi-transparent to the beam. In view of even higher flux densities planned at this beamline, we explored an alternative approach.

## 2. Layout   

Because the direct exposure of the diode to the brilliant beam can cause strong radiation damage, an indirect detection of the beam was implemented.

The beamstop, illustrated in Fig. 1 ▶(*b*), is machined from a single piece of a highly X-ray absorbing tungsten alloy into a chamber with two openings, one for the beam to enter and one for the scattered radiation to exit and hit the photosensitive area of the diode. The outer diameter of the hollow cylindrical part is 2 mm. The back of the cavity is formed by an oblique surface where the beam is absorbed and partly back-scattered. The latter photons can be detected by the diode that is oriented parallel to the beam facing the oblique surface. Scattered photons not directed towards the diode are absorbed in the wall of the tungsten cavity.

For the detecting sensor, a Si photodiode (S1227-16BQ, Hamamatsu) is used (Fig. 1 ▶ *c*). Its elongated rectangular shape and its orientation permit one to minimize the shadow on the detector while providing a relatively large solid angle for the detection of the scattered radiation. The resulting photocurrent is amplified using a low-noise amplifier. During measurements it is essential that any vibration of the beamstop is prevented and its position remains constant. This is achieved by fixing the tungsten cylinder and PIN diode to one end of a profiled and tapered aluminium bar along which the electrical cables can be guided and protected. The bar is connected to a translation system that allows precise positioning from outside the vacuum tube.

## 3. Tests   

**Figure 2:**
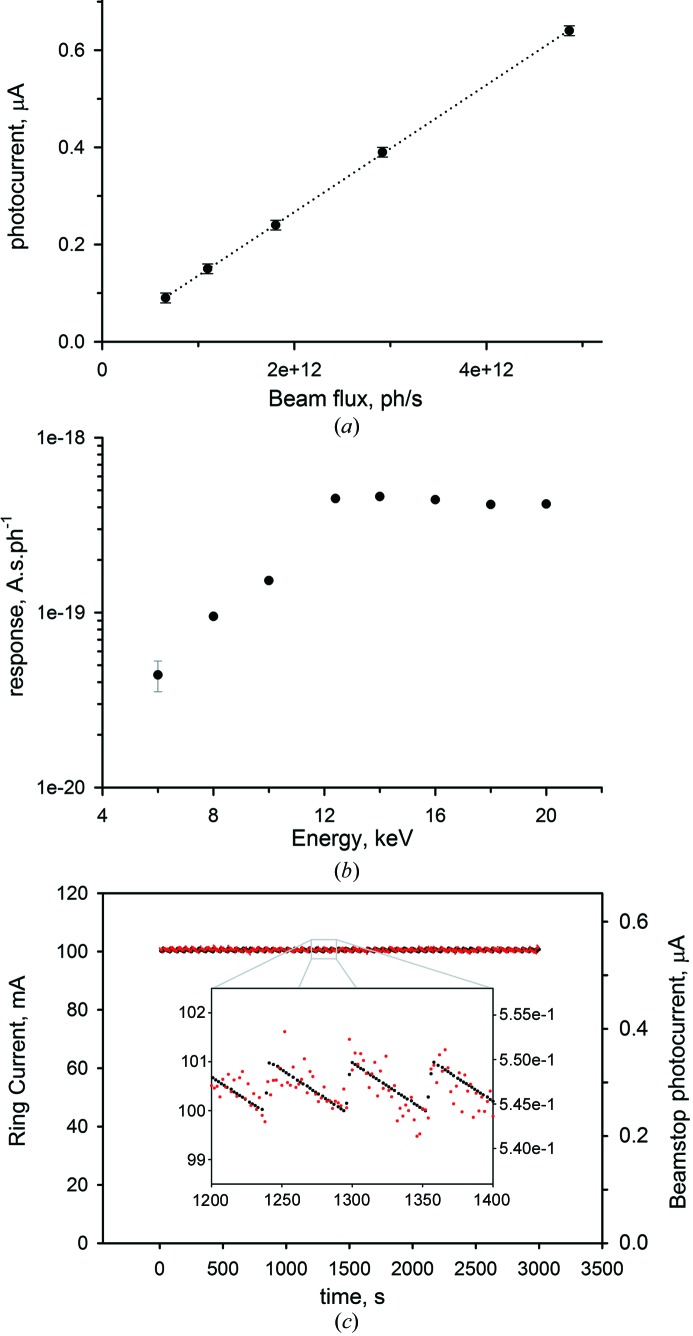
(*a*) Photocurrent *I* produced by the diode as a function of the incident photon flux. (*b*) Response of the active beamstop monitor for different energies (the errors are within the size of the symbol except for 6 keV, measured with a lower photon flux). (*c*) Time series of the beamstop photocurrent (red) and the ring current (black). In the magnified insert the ‘topping up’ operation of PETRA III is reflected in the measured photocurrent.

The beamstop was remotely aligned inside the vacuum tube in front of the detector of the P12 bioSAXS beamline. The proportionality of the beamstop signal with the beam flux was verified using monochromatic 10 keV radiation free from contamination by higher harmonic energies. The incoming intensity was varied by inserting Al foils of different thicknesses. The linearity of the photocurrent with the photon flux is shown in Fig. 2 ▶(*a*), where the photocurrent produced in the diode is plotted as a function of the photon flux measured with a calibrated diode (supplier: IRD; model: AXUV100G), which is used as a secondary standard for intensity measurements at the beamline.

The beamstop was also tested at different energies in the range between 6 and 20 keV. The photocurrent of the diode and the incident flux were measured independently. The ratio between the two quantities as a function of energy is shown in Fig. 2 ▶(*b*), which describes the energy response of the diode. In the high-energy range, between 12 and 20 keV, the active beamstop response shows a nearly constant value of about 4.5 × 10^−19^ A s photon^−1^. At lower energies, the response of the monitor decreases. The sensitivity of the beam intensity measurement is illustrated in Fig. 2 ▶(*c*) displaying the beamstop photocurrent and the ring current over 50 min. Assuming that the beam hitting the beamstop is directly proportional to the quasi noise-free ring current signal, the RMSD is equal to 0.3%. Given that this RMSD value reflects not only the performance of the beamstop but is also influenced by small beam instabilities (*e.g.* vibrations), the intrinsic accuracy of the beamstop is expected yet to be better than 0.3%.

**Figure 3:**
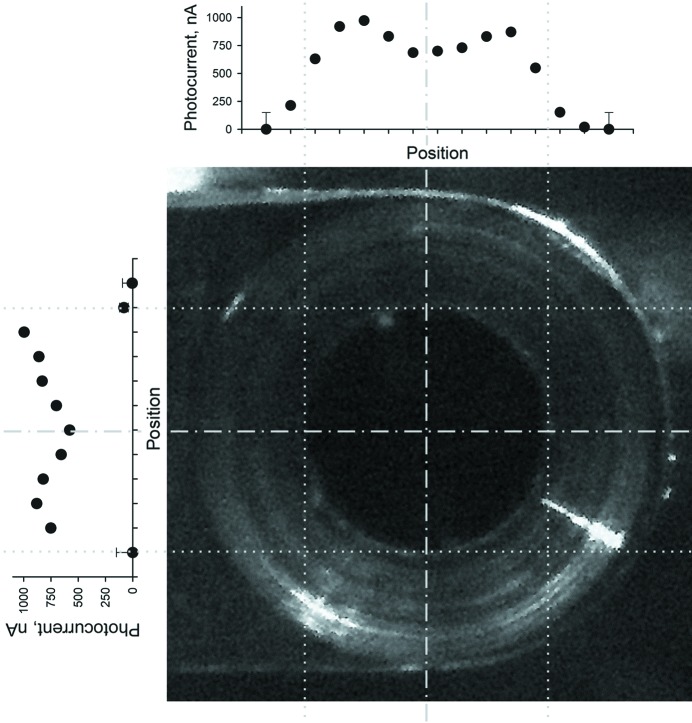
Spatial sensitivity of the beamstop: photocurrent produced by the beamstop for different positions of the beam along the horizontal and the vertical directions.

The response of the beamstop is spatially non-uniform, *i.e.* the photocurrent produced depends on the position of the beam on the beamstop, or, more exactly, the position through which the beam enters the tungsten chamber. The measurement presented so far has been performed with the beam entering the chamber in the middle of the opening. The spatial sensitivity of the beamstop has been characterized at 10 keV by measuring the photocurrent for different positions of the beam along the vertical and the horizontal axes (Fig. 3 ▶). In both directions, the center of the beamstop corresponds to a local minimum of the photocurrent produced by the beamstop. The photocurrent increases when the beam enters the tungsten chamber close to the side of the opening until the beam starts to be cut by the edges of the opening resulting in a decrease of the signal. This decrease is more pronounced in the vertical direction because the beam dimension is smaller along this direction (110 µm *versus* 200 µm FWHM).

## 4. Discussion   

The beamstop described here is particularly robust and maintenance-free because only the massive tungsten part is hit by the intense primary beam. The radiation-sensitive part of the beamstop, the diode, is only exposed to the much less intense scattered radiation and its lifetime can thus be considerably extended.

In order to explain qualitatively the spectral response of the beamstop system, the interaction of X-rays with both the diode and the tungsten chamber should be considered. For the energies between 6 and 10 keV, the response curve of the system (Fig. 2 ▶ *b*) follows the increasing response of the diode until it reaches its maximum, which is defined by the thickness of its depleted zone. At yet higher energies the efficiency of the diode falls off because of the decreasing absorption of the Si sensor. However, this decrease is compensated by jump increases in the fluorescence signal produced by tungsten when the energy of the incident radiation reaches the tungsten *L* edges (10.2, 11.5 and 12.1 keV).

The beamstop keeps a relatively high sensitivity at low energy (<8 keV) and the response is not dependent on the absorption of the material in which the diode might be embedded as it is the case with other types of active beamstops (Ellis *et al.*, 2003 ▶). Moreover, there is no need to attenuate the incident intensity at different X-ray energies to match the dynamic range of the sensor.

Although the sensitivity of the beamstop has been shown to be dependent of the position of the beam with respect to the beamstop (Fig. 3 ▶), the central part of the beamstop signal is rather uniform and robust with respect to displacements that are smaller than the beam size. Note that the variations of the beam position observed during a measurement are much smaller, being a fraction of this value.

The indirect detection approach also allows more flexibility in the design. In commonly used designs, where the beam directly hits the diode, the size of the beamstop is generally limited by the size of the diode itself. Although small active beamstops with a diameter of 1.5 mm have been built using miniature diodes (Ellis *et al.*, 2003 ▶), typically the beamstops are larger (a few mm) in size, defined by the size of commercially available radiation hard diodes. The present design overcomes this limitation. The dimensions of the beamstop described here have been optimized for the bioSAXS P12 beamline, but the size can be further reduced, being only limited by the machining of the tungsten chamber. The rectangular diode housing still masks part of the image and a small sector of the azimuthal angle on the detector is shadowed (Fig. S2). However, the shadow of the diode housing is offset with respect to the beam center. For isotropic scattering, signals close to the beam center can be detected in the segment opposite to the diode housing, such that the range of accessible scattering vectors is not restricted. Moreover, the shadow can be minimized by choosing appropriate diode geometry and orientation: typically with a long diode parallel to the beam, the projection of the housing in the direction of the beam is limited while still providing a relatively large photosensitive area.

## 5. Conclusion   

We have developed an active beamstop where the flux transmitted through the sample is measured indirectly by detecting the photons scattered from a surface hit by the beam. This approach permits the flux to be monitored on a high-brilliance beamline that would rapidly damage the sensor if direct detection was used. Indirect detection also allows the geometrical constraints imposed by conventional diode beamstop layouts to be overcome. The beam flux can be measured accurately over a wide range of energies. The design is of special interest for scattering experiments on isotropic samples and the beamstop was successfully and intensely used during six months of user operation on the P12 bioSAXS beamline without showing any sign of degradation over this period.

## Supplementary Material

Figure S1: Photograph of the miniature diode before and after 12 hours of exposure to the beam. Figure S2: Image collected with the Pilatus 2M Pixel detector.. DOI: 10.1107/S160057751402829X/co5061sup1.pdf

## Footnotes

1 Supporting information for this paper is available from the IUCr electronic archives (Reference: CO5061).
