# Supplementary material for: A small and robust active beamstop for scattering experiments on high-brilliance undulator beamlines
Source: J Synchrotron Radiat. 2015 Feb 4;22(Pt 2):461–4. doi: 10.1107/S160057751402829X (PMC4344362; doi:10.1107/S160057751402829X)
Supplement: Supplementary file 1 [file s-22-00461-sup1.pdf]

JOURNAL OF SYNCHROTRON RADIATION

Volume 22 (2015)

Supporting information for article:

A small and robust active beamstop for scattering experiments on high brilliance undulator beamlines

Clement E. Blanchet, Christoph Hermes, Dmitri I. Svergun and Stefan Fiedler

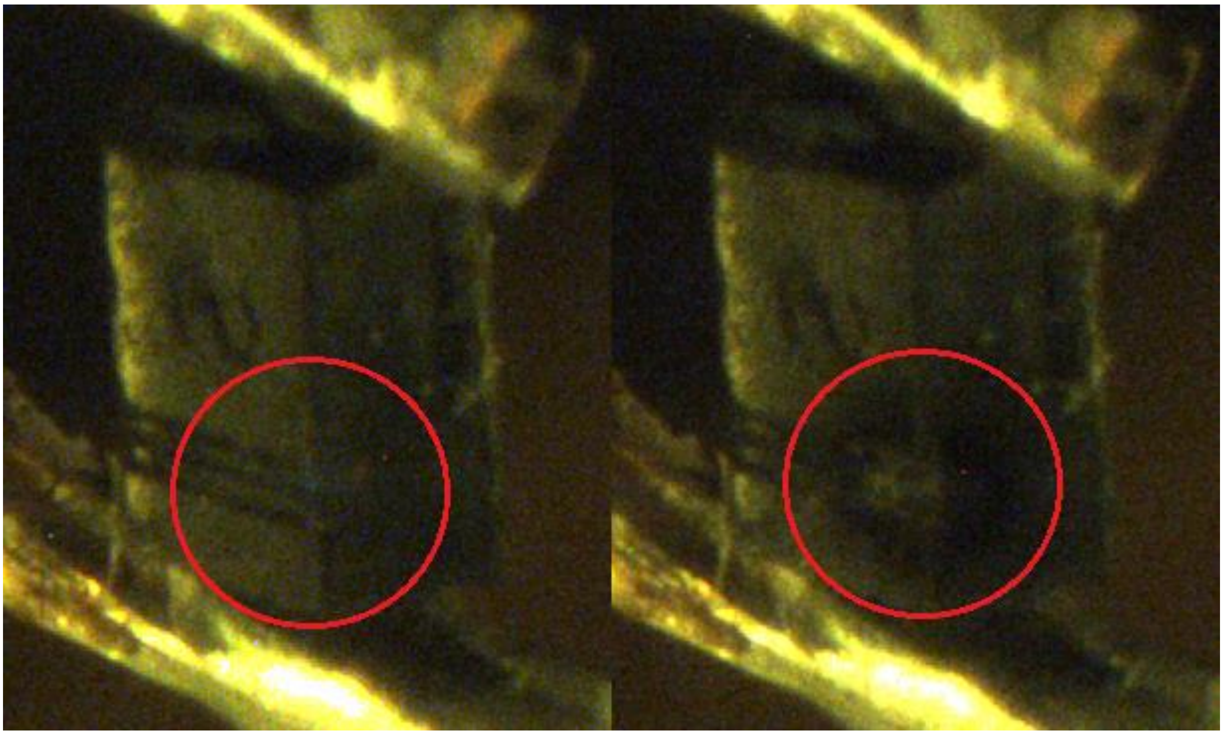

Figure S1 Photo of the miniature diode before and after 12 hours of exposure to the beam. Radiation damage can be recognized inside the red circle.

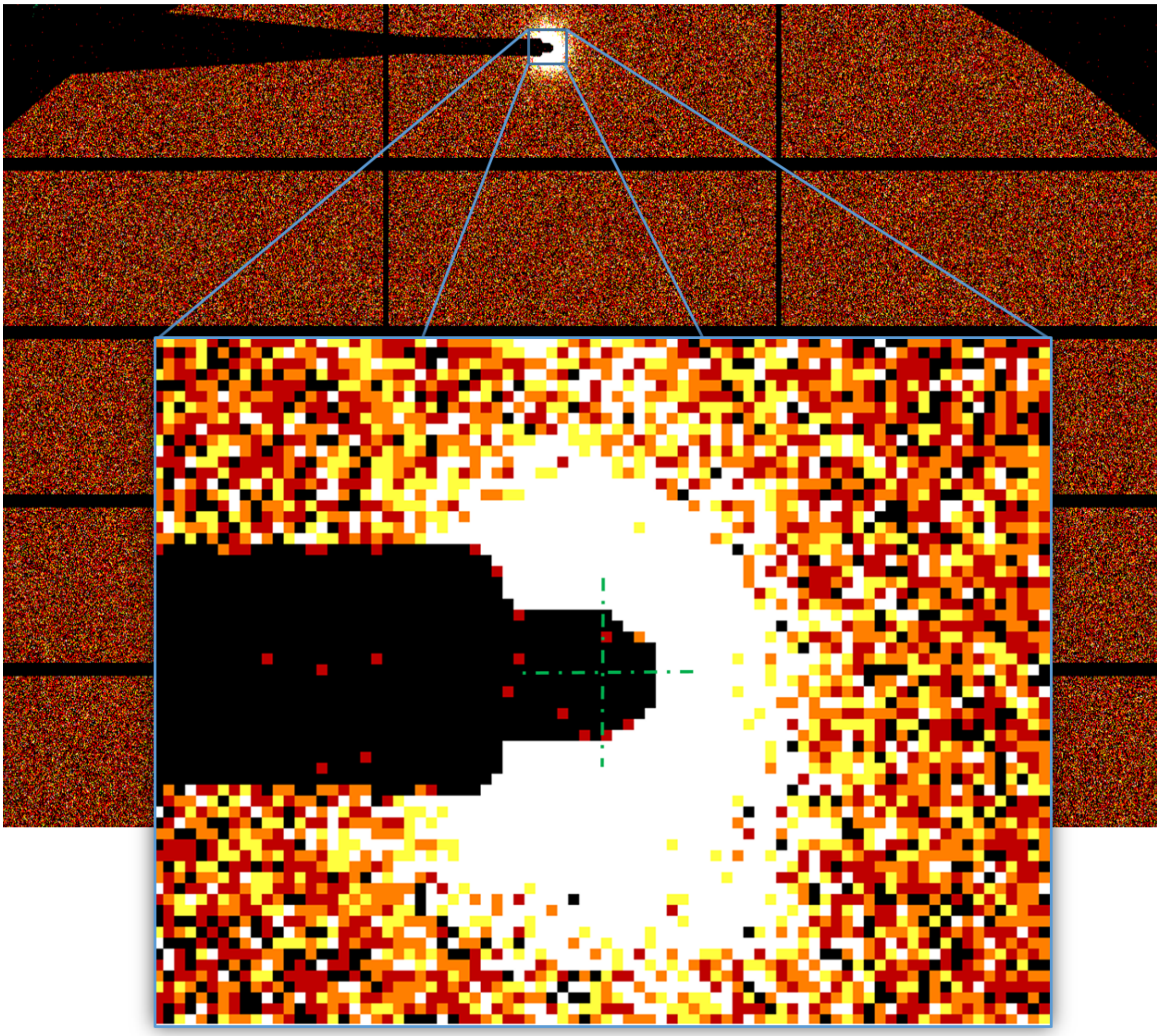

Figure S2 Image collected with the Pilatus 2M Pixel detector (pixel size 172x172µm^2). The inset shows a zoom on the beamstop shadow. The position of the incoming beam is indicated with a green cross.
